# H2AX phosphorylation driven by the SET-PP2A axis maintains glioma stem cell properties

**DOI:** 10.1016/j.gendis.2025.101961

**Published:** 2025-12-02

**Authors:** Chunhua Han, Na Li, Ananya Banerjee, Linzhou Wang, Yajing Yang, Yaogang Zhong, Sophia Wu, Heather Manring, Nan Zhang, Christian A. Showalter, Karnika Singh, Zhen-Yue Tong, Xiaomei Meng, Zaid Sirhan, Jaharul Haque, Qingfei Zheng, Monica Venere, Deliang Guo, Qi-En Wang, Arnab Chakravarti

**Affiliations:** a Department of Radiation Oncology, Comprehensive Cancer Center, The Ohio State University, Columbus, OH 43210, USA; b Columbus Academy, Gahana, OH 43230, USA

**Keywords:** γH2AX, Glioblastoma, Glioma stem cells, H2AX, PP2A, SET

## Abstract

Glioblastoma harbors a subpopulation of glioma stem cells (GSCs) that drive tumor relapse and invasion. Although GSC properties are known to be regulated by key transcription factors and chromatin remodeling, the mechanisms governing stemness-associated gene expression and the role of histone proteins in this process remain poorly understood. Here, we revealed that GSCs possessed an increased level of histone variant H2AX and phosphorylated H2AX at Ser 139 (γH2AX). CRISPR-Cas9-mediated deletion of H2AX demonstrated its critical role in maintaining GSC self-renewal, tumorigenicity, and the expression of multiple stemness-associated genes. Reintroduction of wild-type H2AX, but not the phosphorylation-deficient S139A mutant, restored GSC properties in H2AX-knockout cells, underscoring the importance of H2AX phosphorylation. We further identified that the elevated γH2AX levels in GSCs result from suppressed PP2A phosphatase activity, driven by increased expression of the SET oncoprotein. Pharmacological reactivation of PP2A effectively reduced γH2AX levels in GSCs. Collectively, our findings uncover a novel mechanism for GSC maintenance and highlight the SET-PP2A axis as a key regulator of γH2AX levels, offering a potential therapeutic target to disrupt GSC-driven tumor growth.

## Introduction

Glioblastoma (GBM) is a highly aggressive tumor characterized by frequent recurrence. While standard fractionated radiation is a cornerstone of GBM treatment, it often leads to local relapse and therapy-resistant cell populations. A subset of tumor-initiating cells, commonly termed glioblastoma stem cells (GSCs), has been identified within GBM.^1^^,^^2^ These cells exhibit enhanced tumorigenic capacity, with the ability to self-renew, proliferate, and generate diverse glioma cell populations that contribute to tumor heterogeneity.^3^^,^^4^ Thus, GSCs, along with other cancer stem cells (CSCs), are thought to be key drivers of therapy resistance and tumor recurrence.^2^, ^3^, ^4^, ^5^, ^6^ In addition, glioma cells display significant plasticity, allowing differentiated glioma cells (DGCs) to transition into GSCs in response to various intrinsic and extrinsic factors.^7^, ^8^, ^9^, ^10^ This dynamic interconversion between non-GSCs and GSCs, particularly during and after treatment, replenishes the GSC pool and facilitates tumor regrowth.^11^ The regulation of GSC properties involves key transcription factors and chromatin remodeling processes. Although numerous studies have explored the mechanisms governing GSC maintenance and DGC dedifferentiation,^4^ the precise transcriptional and chromatin-level regulation of stemness remains incompletely understood.

Chromatin structure is crucial for regulating DNA-templated processes, including gene expression. In embryonic stem cells, chromatin organization is uniquely structured, with developmental gene promoters often exhibiting a bivalent state marked by both activating and repressive histone modifications.^12^ This dynamic chromatin landscape plays a key role in maintaining pluripotency, enabling cellular reprogramming, and guiding differentiation, which collectively define the stem cell state.^13^ Similarly, GSCs possess distinct chromatin accessibility patterns^14^ and unique histone modification profiles.^15^ These characteristics enable GSCs to reorganize their chromatin architecture, allowing for flexible gene expression in response to signals from the tumor microenvironment.^15^ Furthermore, chromatin remodeling in GSCs enhances their plasticity and contributes to therapy resistance, promoting adaptation and survival under treatment-induced stress.^16^

In addition to modifications of canonical histones, such as H2A, H2B, H3, and H4, histone variants play pivotal roles in regulating diverse chromatin-associated processes. The histone variant H2AX constitutes approximately 2.5%–25% of the total H2A pool in mammalian cells and features a unique extended C-terminal tail containing a conserved serine–glutamine motif. Following DNA damage, H2AX is phosphorylated at serine 139 (S139), forming γH2AX, a critical early event in the DNA damage response (DDR). This phosphorylation facilitates the recruitment and assembly of a multiprotein complex at sites of double-strand breaks (DSBs), coordinating DNA repair and activating cell cycle checkpoints.^17^ γH2AX is more abundant in embryonic stem cells than in differentiated cells and plays a crucial role in embryonic stem cell proliferation.^18^ Its presence at ribosomal DNA within nucleoli influences self-renewal capacity.^19^ During DDR, γH2AX contributes to chromatin relaxation, ensuring the retention of repair factors at damaged sites and promoting efficient repair. Interestingly, in pluripotent cells, higher basal γH2AX levels correlate with global chromatin decompaction rather than pre-existing DNA damage, indicating its role in sustaining the pluripotent state.^20^, ^21^, ^22^

In this study, we identified heightened basal levels of both H2AX and γH2AX in GSCs compared with their corresponding DGCs. Functional analyses demonstrated that both H2AX and γH2AX are essential for maintaining GSC self-renewal and tumorigenic capacity. Mechanistically, we showed that elevated levels of the SET oncoprotein contribute to increased γH2AX expression by suppressing the protein phosphatase 2A (PP2A) activity, thereby sustaining γH2AX levels critical for GSC maintenance.

## Materials and methods

### Cell lines, patient-derived xenografts, cell culture, and reagents

Human GBM xenograft-derived cell lines GBM387 and GBM3359 were generously provided by Dr. Jeremy Rich (University of Pittsburgh), while GBM43, GBM3691, and GBM10 were obtained from the Mayo Clinic. These cells were maintained as suspension cultures in serum-free GSC medium, consisting of Neurobasal medium supplemented with 20 ng/mL epidermal growth factor (EGF) (R&D Systems), 20 ng/mL fibroblast growth factor (FGF) (R&D Systems), 1 mM sodium pyruvate (ThermoFisher), 2 mM glutamate (ThermoFisher), 1X B27 (ThermoFisher), and 100 U/mL penicillin-streptomycin (Sigma). Established GBM cell lines U251, T98G, and LN229 were sourced from the American Type Culture Collection (ATCC), and the Lenti-X 293T cell line was purchased from Takara. These cell lines were cultured in DMEM supplemented with 10% fetal bovine serum (FBS) and 100 U/mL penicillin-streptomycin. All cell lines were maintained in a humidified incubator at 5% CO_2_ and were routinely authenticated using short tandem repeat (STR) DNA profiling and tested for mycoplasma contamination. For differentiation induction, GBM xenograft-derived cells were cultured on Geltrex-coated dishes (ThermoFisher) with the addition of 10% FBS or 50 ng/mL BMP4 (STEMCELL Technologies) for two weeks. To promote dedifferentiation, U251, T98G, and LN229 cells were grown in suspension using ultra-low attachment dishes with standard serum-free GSC medium for two weeks.

### Plasmids, shRNA, gene transfection, and lentivirus production

The pcDNA6.2/EmGFP/H2AX-WT and pcDNA6.2/EmGFP/H2AX-S139A plasmids were generally provided by Dr. Justin Leung (University of Texas Health Science Center at San Antonio). CRISPR sgRNA targeting human H2AX (H2AX-sg1: 5′-CTCGCTAGCATGTCGGGCCG-3′; H2AX-sg2: 5′-CAAGAAGACCAGCGCCACCG-3′) were designed and inserted into pLentiCRISPR v2 all-in-one plasmids by Genscript. shRNA constructs targeting SET were obtained from Millipore Sigma (shSET-1: 5′- GCGATTGAACACATTGATGAA-3′) and VectorBuilder (shSET-2: 5′-CCACCGAAATCAAATGGAAAT-3′). Cells were either transfected with plasmids using Lipofectamine 2000 reagent (ThermoFisher, Cat. #11668019) following the manufacturer's protocol or infected with lentivirus.

For lentivirus production, 4 × 10^6^ Lenti-X 293T cells were plated in a 100 mm dish and co-transfected with three plasmids: the lentiviral vector (8 μg), the packaging plasmid (psPAX2, 6 μg), and the envelope plasmid (pMD2.G, 4 μg), using Lipofectamine 2000 (ThermoFisher). After 48–72 h, the culture medium was collected and centrifuged at 500 *g* for 5 min. The supernatant was transferred to a clean tube, and lentiviral particles were concentrated using the Lenti-X concentrator (TaKaRa, Cat. #631232) before being resuspended in phosphate-buffered saline solution (PBS).

### CRISPR-Cas9-based gene knockout

To generate stable H2AX knockout (KO) cell lines. GBM cells were transduced with lentiviral pLentiCRISPR all-in-one plasmids carrying H2AX-targeting sgRNA. After 48 h, puromycin (2 μg/mL) was added to select for successfully transduced cells. Surviving cells were then seeded into 96-well plates at limiting dilution to enable the isolation and expansion of single-cell clones. The knockout efficiency was validated in both pooled transduced populations and individual clones using Western blot analysis.

### Mass spectrometry-based proteomics

To analyze differential histone protein expression between GSCs and DGCs, GBM387 GSCs and their serum-induced DGC counterparts were lysed in a buffer containing 5% SDS and 50 mM triethylammonium bicarbonate. After centrifugation, the supernatant was collected. Protein samples (75 μg) were reduced using DTT, alkylated with iodoacetamide, and enzymatically digested on a Strap mini column (ProtiFI) with LysC and trypsin (1.5 μg each, Promega) at 37 °C overnight. The resulting peptides were eluted, reconstituted in 0.1% formic acid, and subjected to nano LC-MS/MS analysis on a Thermo Eclipse mass spectrometer equipped with a FAIMS DUO Pro source, coupled to a nanoElute LC system (ThermoFisher). Mass spectrometry data were processed using MASCOT and searched against the human UniProt database (20,421 reviewed entries).

To further identify differentially expressed proteins in GSCs and DGCs, GBM387 and GBM3359 GSCs, along with their serum-induced DGC counterparts, were lysed in RIPA buffer, followed by centrifugation to collect the supernatant. Protein samples (10 μg) were precipitated using trichloroacetic acid (TCA) at a 1:3 ratio and resuspended in a denaturing solution containing 8 M urea and 100 mM Tris–HCl (pH 8.5). Proteins were reduced with 5 mM Tris (2-carboxyethyl)phosphine (TCEP), alkylated with 10 mM iodoacetamide (IAA), diluted with Tris–HCl, and digested overnight at 37 °C with 0.5 μg trypsin. Peptides were desalted using a C18 column, dried under vacuum, and reconstituted in 0.1% formic acid. For mass spectrometry analysis, 200 ng of peptides from each sample were injected into a Bruker timsTOF Pro mass spectrometer equipped with a CaptiveSpray ion source and coupled to a nanoElute LC system (Bruker). The raw MS and MS/MS data were analyzed using Fragpipe software (v21.1) and searched against the human UniProt database. Differentially expressed proteins were identified using *t*-test analysis with a fold-change threshold of > 2 or < 0.5 and a significance cutoff of *P* < 0.05, with multiple testing correction applied using the Bonferroni method.

### Western blotting analysis

Whole-cell lysates were prepared using an SDS lysis buffer containing 2% (w/v) SDS, 10% (v/v) glycerol, 62 mmol/L Tris–HCl (pH 6.8), and a complete protease inhibitor cocktail (Roche Applied Science). Protein concentrations were determined using the DC Protein Assay (Bio-Rad). Equal protein amounts were separated via SDS-PAGE and transferred onto a nitrocellulose membrane. The membrane was blocked with 5% nonfat milk in Tris-buffered saline-Tween 20 and incubated overnight at 4 °C with the following primary antibodies: Rabbit anti-SOX2 (CST, Cat. #3579S, 1:1,000), Rabbit anti-OLIG2 (CST, Cat. #2748, 1:1000), Rabbit anti-NANOG (CST, Cat. #4903, 1:1,000), Mouse anti-GFAP (BioLegend, Cat. #644702, 1:1,000), Rabbit anti-H2AX (CST, Cat. #7631, 1:1,000), mouse anti-γH2AX (Millipore Sigma, Cat. #05-636, 1: 2,000), Rabbit anti-Tubulin (CST, Cat. #2144, 1:1,000), Rabbit anti-GAPDH (CST, Cat. #2118, 1:1,000), Rabbit anti-ATM (CST, Cat. #2873, 1:1,000), Rabbit anti-pATM (S1981) (CST, Cat. #5883, 1:1,000), Rabbit anti-ATR (CST, Cat. #13934, 1:1000), Rabbit anti-pATR (S428) (CST, Cat. #2853, 1:1000), Rabbit anti-pRPA32 (S33) (CST, Cat. #10148, 1:1,000), Rabbit anti-RPA32 (CST, Cat. #35869, 1:1,000), Rabbit anti-SET/TAF1 antibody (ABclonal, Cat. #A9173, 1:500), mouse anti-I2PP2A (SET) antibody (Santa Cruz Biotechnology, Cat. #sc-133138, 1:500). Following multiple washes, the membrane was incubated for 1 h at room temperature with HRP-conjugated goat anti-rabbit or goat anti-mouse secondary antibodies (Millipore Sigma, Cat. #12-348, 12-349). Protein bands were detected using chemiluminescence. Band intensities were quantified using ImageJ (National Institutes of Health), and relative protein levels were normalized to the control group, which was set to a value of 1.

### Flow cytometry and cell sorting

GBM cells were trypsinized, resuspended in PBS containing 1% bovine serum albumin, and stained live with APC-conjugated human CD133/1 antibody (Miltenyi Biotec, Cat. #130-110-963) following the manufacturer's recommended concentration. Appropriate isotype controls were included to define negative gating. CD133^+^ and CD133^–^ cell populations were sorted using a BD Aria III Flow Cytometer and promptly lysed in the SDS lysis buffer for subsequent protein extraction and Western blot analysis.

### RNA sequencing

Total RNA was extracted from U251 wild-type (WT) cells and three clones of U251 cells transfected with H2AX sgRNA (H2AX-sg1 clones C4, C7, and C11; and H2AX-sg2 clones C1, C2, and C3) using the Norgen Total RNA Purification Kit (Norgen Biotek) following the manufacturer's protocol. After quality control, mRNA was isolated from total RNA using poly-T oligo-attached magnetic beads. The mRNA was then fragmented, and first-strand cDNA was synthesized using random hexamer primers. A cDNA library was constructed and sequenced on Illumina platforms by Novogene. The Illumina HiSeq raw image data was converted to Sequenced Reads using the CASAVA base recognition. The raw data were organized into FASTQ files, and low-quality reads or reads containing adapters were removed. The remaining reads were aligned to the hg38 reference genome using the HISAT2 algorithm. Gene expression levels were calculated based on transcript abundance and presented as Fragments Per Kilobase of transcript sequence per Million (FPKM). Differential gene expression analysis was performed using DESeq to identify genes with significant expression changes under different conditions.^23^

### Quantitative reverse transcription PCR

Total RNA was isolated from GBM cells using Trizol reagent (Life Technologies, Carlsbad, California, USA) following the manufacturer's instructions. First-strand cDNA was synthesized using the reverse transcription system (Applied Biosystems) in a 20 μL reaction containing 1.5 μg of total RNA. A 0.5 μL aliquot of cDNA was then amplified using Fast SYBR Green PCR Master Mix (Applied Biosystems) in a 20 μL reaction on a QuantStudio 3 system (Applied Biosystems). Expression levels were normalized to those of 18S rRNA. The primer sequences for PCR are as follows: JAK3: forward: 5′- AGACAGAGGTGCTGCTGAAGGT-3′, reverse: 5′-CGGTACGACACTTGGCTCATCAA-3′; SALL4: forward: 5′-AACCACATCCTTCCAGGCACTCT -3′, reverse: 5′-GACTGCTCCGACCTTCCATCTCA-3′; CXCL12: forward: 5′-TTGTGATTGCCTCTGAAGCCTATGT-3′, reverse: 5′-ATTGGAACCTGAAACCCTGCTGTG-3′; KLF4: Forward: 5′-GCCGCTCCATTACCAAGGTCAG-3′, reverse: 5′-ATCATCCCGTGTGTCCCGAAGT-3′; 18S: forward: 5′-CGTTGATTAAGTCCCTGCCCTT-3′, reverse: 5′-TCAAGTTCGACCGTCTTCTCAG-3′.

### Cell proliferation assay

A total of 2000 GBM387 cells and their H2AX-KO counterparts were seeded in Geltrex-coated 96-well plates and cultured in Neurobasal medium supplemented with EGF (20 ng/mL), FGF (20 ng/mL), sodium pyruvate, glutamate, and B27 for up to 5 days. Similarly, 2000 U251 cells and their H2AX-KO counterparts were seeded into 96-well plates and cultured in DMEM with 10% FBS for up to 5 days. Cell proliferation was measured every 24 h using the CellTiter-Glo 3D Cell Viability Assay (Promega, Cat. #G9681). All data were normalized to day 1 and expressed as relative cell proliferation rates.

### Neurosphere formation assay

To assess neurosphere formation capacity, 2000 GBM cells were plated in ultra-low attachment 6-well plates and cultured in Neurobasal medium with EGF (20 ng/mL), FGF (20 ng/mL), sodium pyruvate, glutamate, and B27 for 10 days. The number of neurospheres formed was counted, and the neurosphere formation rate was calculated. In addition, neurosphere formation was evaluated using *in vitro* limiting dilution. U251 cells, U251-H2AX-sg1 cells transfected with empty vector, H2AX-WT, or H2AX-S139 were plated in ultra-low attachment 96-well plates at decreasing densities (100, 50, 25, and 10 cells per well). Cells were cultured in Neurobasal medium with EGF (20 ng/mL), FGF (20 ng/mL), sodium pyruvate, glutamate, and B27 for 10 days. The number of neurospheres in each well was recorded, and extreme limiting dilution analysis was conducted using the ELDA software (http://bioinf.wehi.edu.au/software/elda), as described previously.^23^^,^^25^

### PP2A activity determination

PP2A phosphatase activity was measured using the PP2A Immunoprecipitation Phosphatase Assay Kit (Millipore-Sigma, Cat. #17-313). Briefly, whole-cell extracts were prepared using a lysis buffer containing 20 mM imidazole-HCl, 2 mM EDTA, 2 mM EGTA, and protease inhibitors (10 μg/mL each of aprotinin, leupeptin, pepstatin, and 1 mM benzamidine, and 1 mM PMSF). The lysates were incubated overnight at 4 °C with pNPP Ser/Thr Assay Buffer, anti-PP2Ac antibody, and Protein A agarose slurry. After washing the beads, protein phosphatase activity was assessed by adding 750 μM phosphopeptide substrate and incubating at 30 °C for 10 min. The reaction was stopped by adding Malachite Green Phosphate Detection Solution, and phosphatase activity was measured by optical density at 650 nm.

### Comet assay

The comet assay was conducted using the Comet Assay Kit (RD Systems, Cat. #4250-050-K) to assess DNA DSBs. Cells (1 × 10^5^/mL) were mixed with Comet LM Agarose at a 1:10 ratio (50 μL cells with 500 μL agarose), and 50 μL of the mixture was quickly placed onto each well of a Comet Slide, which was allowed to solidify flat at 4 °C in the dark for 10 min. The slides were then submerged in lysis solution at 4 °C overnight, followed by a 20-min incubation in freshly prepared alkaline unwinding solution at room temperature. Electrophoresis was performed at 21 V for 30 min. After electrophoresis, the slides were washed twice in water for 5 min each and then in 70% ethanol for 5 min. The slides were air-dried at 37 °C for 10–15 min, stained with 100 μL of diluted SYBR Green for 30 min in the dark at room temperature, rinsed in water, and dried completely at 37 °C. DNA damage was observed using the revolve fluorescent microscope (ECHO).

### Immunofluorescence

Immunofluorescence staining was carried out following a previously established protocol.^24^ In brief, GBM cells were plated onto Geltrex-coated coverslips and cultured for 24 h. The cells were then fixed with 4% paraformaldehyde and permeabilized with 0.1% Triton X-100. After blocking with 20% normal goat serum in PBS, the coverslips were incubated overnight at 4 °C with the mouse anti-γH2AX antibody (Millipore Sigma, Cat. #05-636, 1:200). Following four washes with phosphate-buffered saline-Tween 20 (PBST), the cells were incubated at room temperature for 1 h with FITC-conjugated goat anti-mouse IgG. After additional washing with PBST, the slides were mounted with Vectashield mounting medium containing DAPI (Vector Laboratories) and visualized using the Revolve fluorescent microscope (ECHO).

### Mouse experiments

The animal experiments described in this manuscript were approved by the Institutional Animal Care and Use Committee (IACUC) at the Ohio State University. All procedures were conducted in accordance with the relevant regulatory standards and guidelines for the ethical treatment of animals. Six to eight-week-old nude mice were obtained from The Jackson Laboratory. To assess tumor-initiating cell frequency (TICf) using a limiting dilution assay, specified cell numbers (1:1 mixture with Matrigel Matrix, Corning, Cat. No. 356231, 0.1 mL total volume) were subcutaneously injected into the axilla of male and female nude mice (1:1 ratio). Tumor formation was monitored for up to 4 weeks post-injection, and the number of tumors in each group was recorded to calculate TICf using the Extreme Limiting Dilution Analysis (ELDA) software (http://bioinf.wehi.edu.au/software/elda/indew.html).^25^

For the intracranial orthotopic model, as previously described,^26^^,^^27^ 1 × 10^5^ GBM387 cells, GBM387-H2AX-sg1, or GBM387-H2AX-sg2 cells were suspended in 2 μL of HBSS and stereotactically injected into the lateral ventricles of female nude mice. Following the injection, the mice were monitored daily for any neurological changes or a body weight loss exceeding 20%, at which point they were humanely euthanized.

### Statistical analysis

Data were presented as mean ± standard deviation in all figures, including histograms and line plots. For comparisons between two groups, two-sample *t*-tests were employed, while ANOVA was used for comparing multiple groups. Reverse transcription PCR data were normalized to the internal control and analyzed using the 2^ΔCT^ method. Holm's procedure was applied to control the family-wise error rate for multiple comparisons. The TICf analysis was performed using the generalized linear model (GLM) outlined by Hu and Smyth.^25^ Kaplan–Meier survival curves were used to evaluate the survival of xenograft-bearing mice, with group differences assessed via the log-rank test. All statistical tests were two-sided, and a significance level of *P* < 0.05 was used.

## Results

### Glioma stem cells possess increased base levels of H2AX and γH2AX

**Fig. 1:**
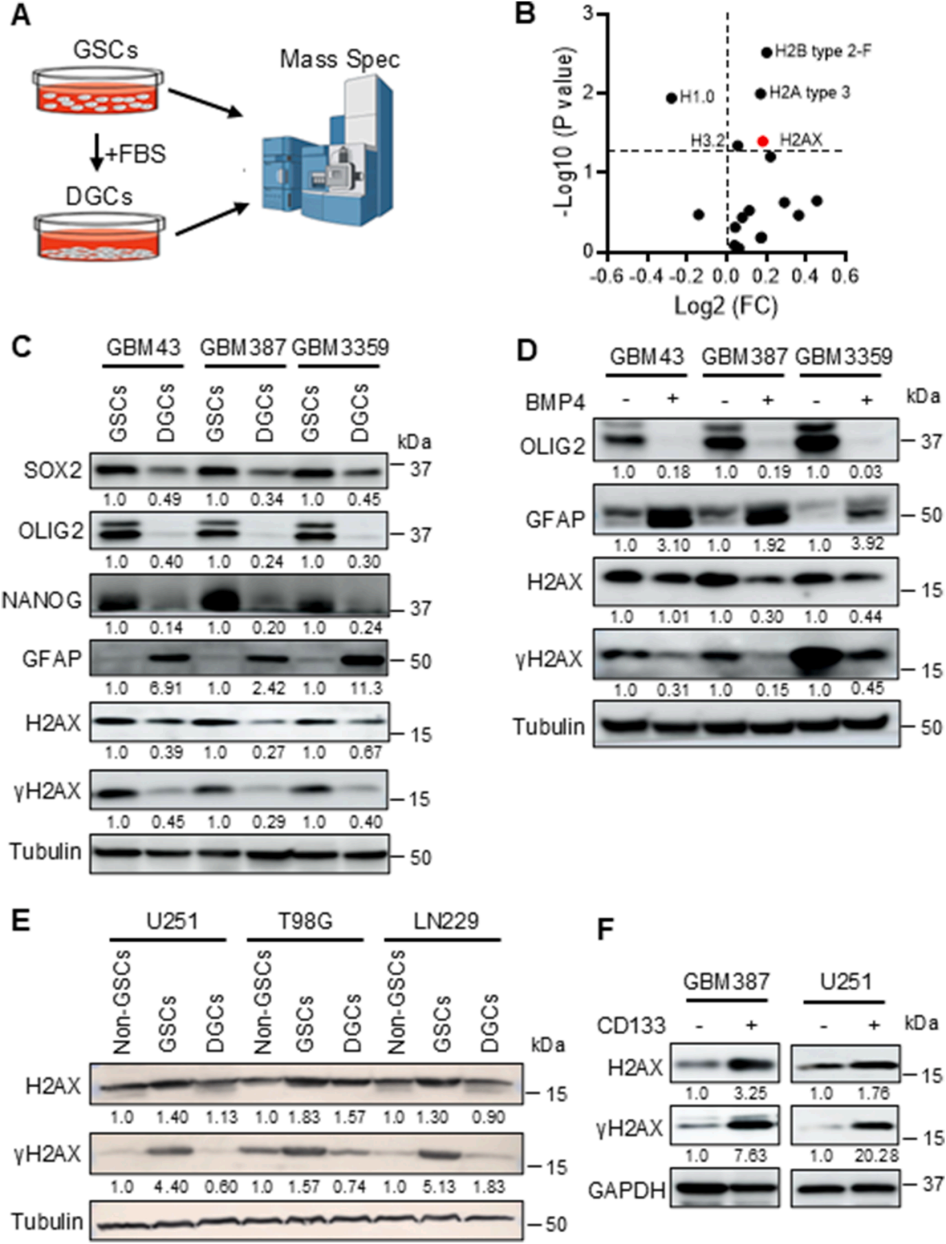
Glioma stem cells (GSCs) possess increased base levels of H2AX and γH2AX. **(A, B)** Proteomics analysis of GSCs and their corresponding differentiated glioma cells (DGCs). GBM387 cells were cultured under spheroid culture conditions to maintain their stemness properties (GSCs). Cells were also cultured in the presence of 10% FBS to induce differentiation (DGCs). Proteomics analysis was performed to identify differentially expressed proteins, with a primary focus on histones (A). Differentially expressed histones are shown in the volcano plot (B). **(C, D)** Validation of the differentially expressed H2AX and γH2AX in GSCs and DGCs. Immunoblotting was conducted to evaluate the expression levels of H2AX and γH2AX in three lines of GBM xenograft-derived GSCs and their corresponding FBS-induced DGCs (C) or BMP4-induced DGCs (D). A panel of stemness genes was also assessed to validate the stemness characteristics of GSCs compared with DGCs. **(E)** Expression levels of H2AX and γH2AX in GBM cell lines cultured under adherent conditions compared with their corresponding spheroid cultures enriched for GSCs. U251, T98G, and LN229 cells were initially cultured under adherent conditions with FBS to preserve the non-GSC population. To enrich for GSCs, the cells were cultured in Neurobasal medium supplemented with B27, EGF, and bFGF under suspension conditions. Following enrichment, the GSCs were returned to adherent culture with serum to generate DGCs. Immunoblotting was performed to evaluate the expression levels of H2AX and γH2AX. **(F)** Expression levels of H2AX and γH2AX in GSCs characterized by CD133^+^ compared with their corresponding CD133^–^ cells. CD133^–^ and CD133^+^ cells were sorted from GBM387 and U251 cells. H2AX and γH2AX were examined in these cells immediately after sorting. Each band was scanned and its intensity quantified; the relative intensity values are shown below the corresponding bands in the figure.

The regulation of histone levels and the incorporation of specific histone variants are critical for shaping the epigenetic landscape that supports the maintenance, plasticity, and therapy resistance of CSCs. To identify dysregulated histones and histone variants in GSCs compared with DGCs, we cultured the primary GBM xenograft-derived GBM387 cells in neurobasal medium supplemented with B27, EGF, and basic fibroblast growth factor (bFGF) under suspension culture conditions to enrich for GSCs.^28^^,^^29^ In parallel, the same cells were cultured in medium supplemented with 10% FBS under adherent conditions for 12 days to generate DGCs.^9^ Subsequently, LC-MS/MS was used to analyze the protein expression profiles of both GSCs and DGCs (Fig. 1A). We identified 16 histones and histone variants, among which 4 (H2B type 2-F, H2A type 3, H2AX, and H3.2) exhibited significantly higher expression levels in GSCs compared with DGCs, while one histone protein (H1.0) showed lower expression in GSCs (*P* < 0.05) (Fig. 1B; Table S1).

It has been shown that H2AX is highly expressed in undifferentiated mouse embryonic stem cells^30^ and plays a crucial role in embryogenesis.^31^ In addition, γH2AX has been reported to be critical for maintaining the self-renewal of pluripotent stem cells and for inducing a pluripotent state during cellular reprogramming.^21^^,^^22^ In light of these findings, we sought to further investigate the functional role of H2AX and γH2AX in the maintenance of GSCs. To validate the heightened levels of H2AX observed in the proteomics analysis, we cultured primary GBM43, GBM387, and GBM3359 cells under spheroid culture conditions to enrich for GSCs. We also added FBS to the adherent culture to generate DGCs. The expression levels of stem cell markers, such as SOX2, OLIG2, and NANOG, were found to be higher in GSCs than in DGCs, while the level of the differentiation marker GFAP was lower in GSCs than in DGCs, confirming the stemness traits in GSCs vs DGCs (Fig. 1C). We indeed observed elevated levels of H2AX in GSCs compared with DGCs. Furthermore, γH2AX levels were also higher in GSCs (Fig. 1C). In addition to using serum to induce GSC differentiation, we also induced GSC differentiation with BMP4.^28^ Again, we found heightened levels of H2AX and γH2AX in GSCs compared with DGCs (Fig. 1D).

Established GBM cell lines, including U251, T98G, and LN229, are typically cultured adherently in medium supplemented with 10% FBS to maintain a differentiated status. To enrich for GSCs, we cultured these cell lines under the aforementioned GSC culture conditions (sphere), followed by differentiation induction in medium with 10% FBS under adherent conditions (differentiation). We observed a clear increase in both H2AX and γH2AX levels in the enriched GSCs, which decreased upon differentiation (Fig. 1E). To rule out the possibility that culture conditions might influence the protein levels of H2AX and γH2AX, we sorted CD133^+^ and CD133^-^ cells from GBM387 and U251 cell lines, representing GSCs and non-GSCs, respectively,^32^ and examined the expression of H2AX and γH2AX immediately. CD133^+^ cells showed significantly elevated levels of both H2AX and γH2AX (Fig. 1F). Additionally, these findings were corroborated in two other primary GBM cell lines (Fig. S1A and S1B). In summary, all these results indicate that GSCs possess increased levels of total H2AX and γH2AX. Furthermore, elevated H2AX and γH2AX levels were also found in another two primary glioblastoma cells, and the increased γH2AX levels in GSCs were confirmed by immunofluorescence (Fig. S1C and S1D). In conclusion, these results indicate that GSCs exhibit elevated levels of both total H2AX and γH2AX.

### H2AX is critical to the maintenance of GSCs

**Fig. 2:**
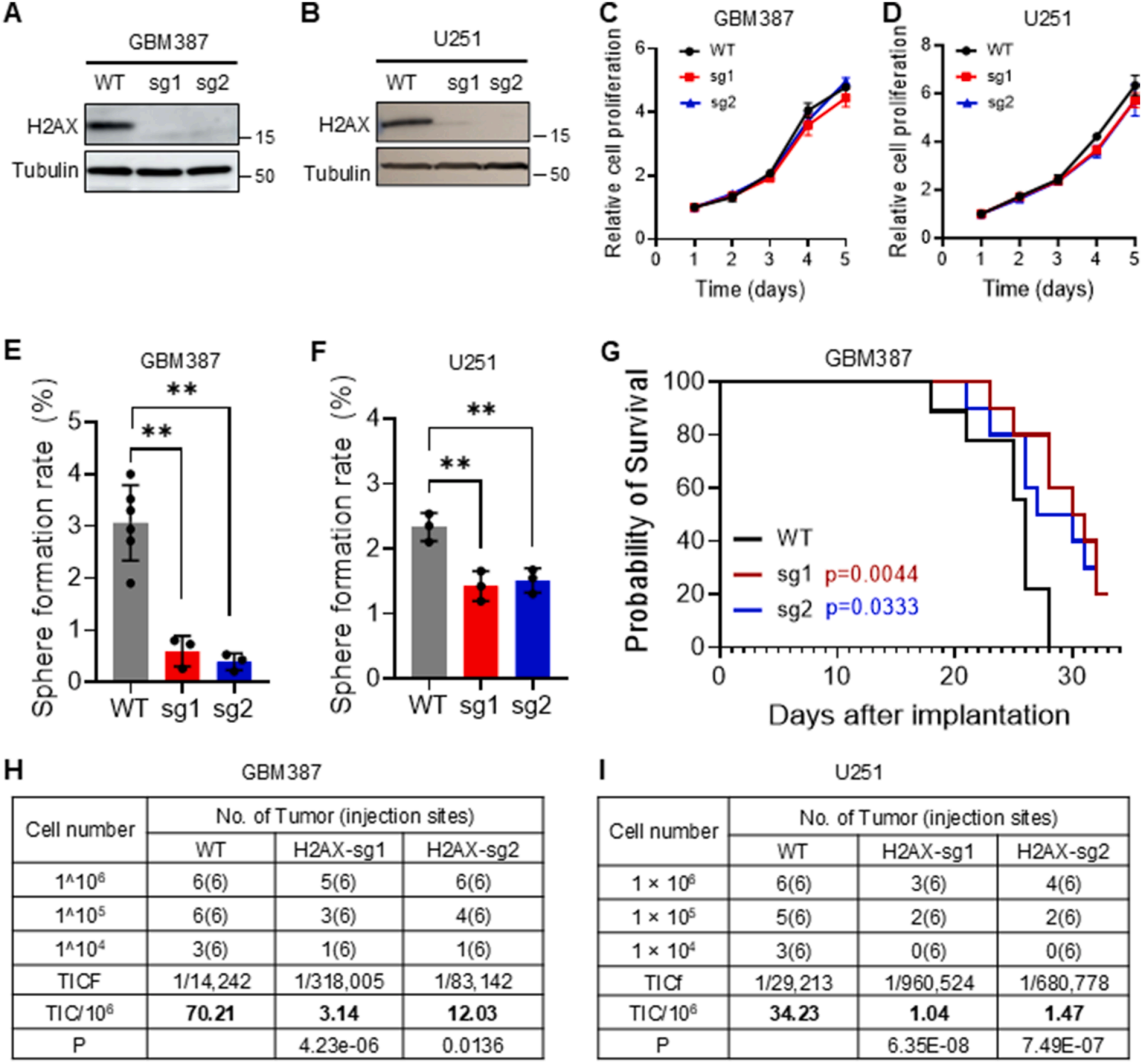
H2AX is critical to the maintenance of glioma stem cells. **(A, B)** H2AX knockout (KO) cell lines were established in GBM387 (A) and U251 (B) cells using two distinct sgRNA. **(C, D)** Cell proliferation was assessed using methylene blue assays. *n* = 4; bar: standard deviation. **(E, F)** Neurosphere formation capacity was evaluated using sphere formation assays in H2AX knockout GBM387 (E) and U251 (F) cells. bar: standard deviation. ∗∗*P* < 0.01. **(G)** Kaplan–Meier survival curves of immunocompromised mice implanted intracranially with GBM387 cells, comparing survival outcomes between mice bearing wild-type (WT) and H2AX knockout tumors (*n* = 9 for WT; *n* = 10 for sg1 and sg2). **(H, I)** The frequency of tumor-initiating cells (TICf) was determined via a xenotransplantation limiting dilution assay. GBM cells were injected subcutaneously into nude mice at three different cell doses (1 × 10^6^, 1 × 10^5^, and 1 × 10^4^) per group, with an equal male-to-female ratio (1:1). Tumor formation was monitored for 2 weeks post-injection, and the number of tumors formed at each dose was recorded. TICf was then calculated using Extreme Limiting Dilution Analysis (ELDA) software.

To investigate the role of H2AX in the maintenance of GSCs, we generated H2AX KO cell lines in U251 and GBM387 cells using CRISPR-Cas9 (Fig. 2A and B). First, we assessed the impact of H2AX KO on the proliferation of GBM cells and found that targeting H2AX did not impair proliferation in GSCs (Fig. 2C and D). Next, we examined the self-renewal ability of these cells using the neurosphere formation assay. We found that H2AX KO significantly reduced the ability of GBM cells to form spheres under spheroid culture conditions (Fig. 2E and F; Fig. S2A and S2B). Since the capacity to form tumors after xenotransplantation in immunocompromised mice is a key feature of CSCs,^33^ we evaluated the effect of H2AX on the tumorigenicity of GSCs in an intracranial xenograft model. GBM387 and H2AX-KO GBM387 cells were injected intracranially into immunocompromised mice. Kaplan–Meier survival analysis revealed that H2AX KO improved the survival of xenograft-bearing mice (Fig. 2G), suggesting that H2AX plays a critical role in mediating the tumorigenicity of GSCs. We further determined the tumor-initiating cell frequency (TICf) in multiple GBM cell lines with H2AX KO using the xenograft limiting dilution assay.^26^^,^^34^ We found that H2AX depletion decreased the tumorigenicity of all types of GBM cells (Fig. 2H and I; Fig. S2C–S2F), indicating that H2AX is critical to the maintenance of the GSC population in GBM. Taken together, these results indicate that H2AX plays an important role in maintaining the stemness properties of GBM cells.

### H2AX KO suppresses stemness-related gene expression

**Fig. 3:**
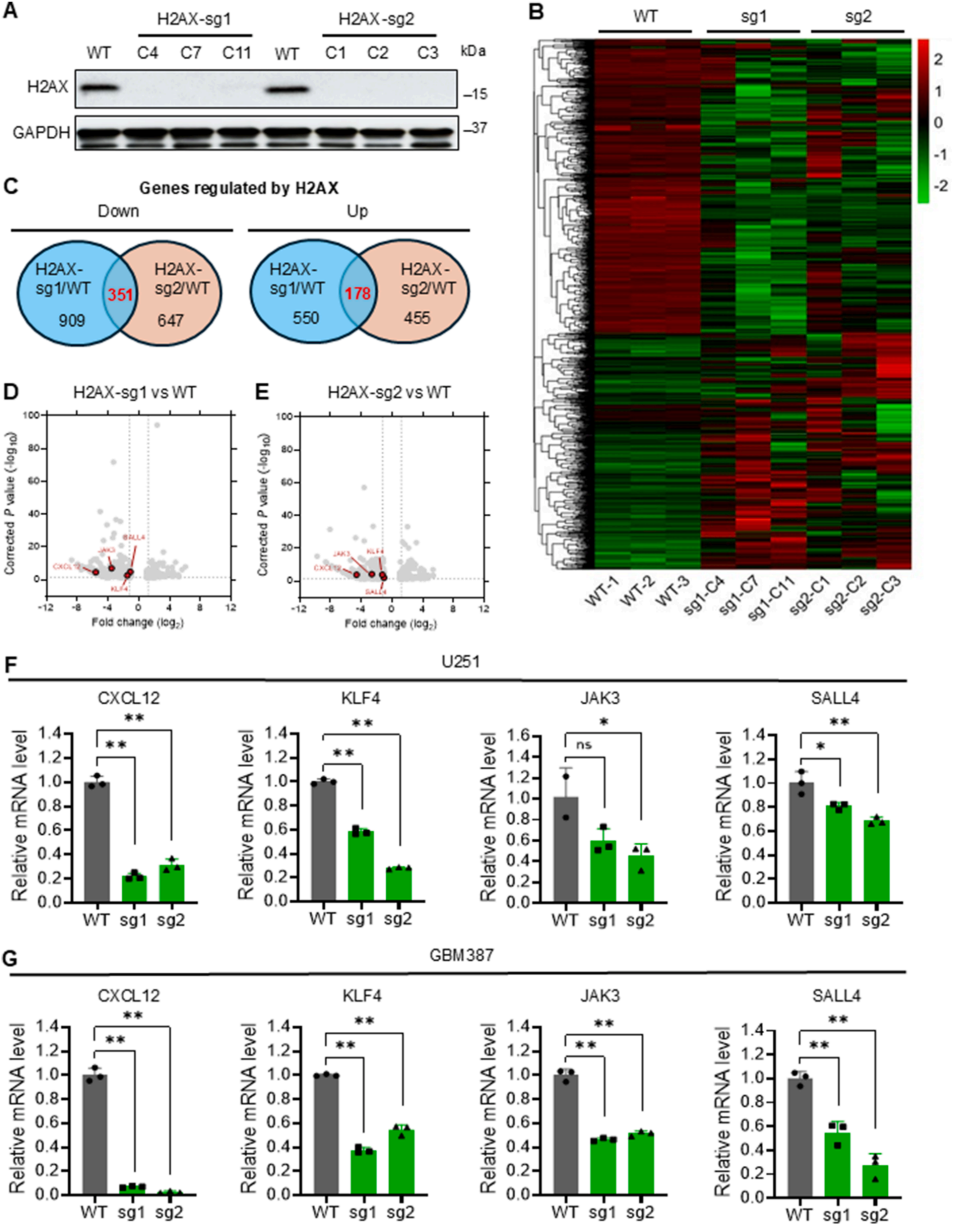
H2AX knockout down-regulates stemness-related gene expression. **(A)** Three single clones were selected from U251 cells transfected with each of the two sgRNA targeting H2AX, sg1, and sg2. **(B**–**E)** Differentially expressed genes were identified in H2AX-KO cells. RNA-sequencing analysis was conducted on three H2AX-sg1 clones, three H2AX-sg2 clones, and U251 parental cells. (B) Heatmap of clustered differentially expressed genes, with red indicating up-regulation and blue indicating down-regulation in H2AX-KO cells compared with H2AX-WT cells. (C) Venn diagram of commonly dysregulated genes (|Log fold-change| > 1; *P* < 0.05) shared between the sg1 and sg2 groups. (D, E) Representative stemness-associated genes significantly down-regulated in H2AX-KO cells. **(F, G)** Validation of the down-regulation of stemness-related genes in H2AX-KO cells using quantitative reverse transcription PCR in U251 (F) and GBM387 (G) cells. *n* = 3; bar: standard deviation; ∗*P* < 0.05 and ∗∗*P* < 0.01.

To further explore the mechanisms through which H2AX facilitates the maintenance of stemness properties in GBM cells, we performed RNA-sequencing analyses to profile gene expression in H2AX-KO U251 cells and compared them with parental U251 cells. Two distinct sgRNAs targeting different regions of H2AX were used to generate H2AX-KO cell lines, and three clones per sgRNA were selected for RNA-sequencing analysis to minimize the effect of clonal variability (Fig. 3A). The RNA-sequencing analysis revealed that H2AX KO significantly altered the expression of numerous genes (Fig. 3B). Specifically, 351 genes were down-regulated, and 178 genes were up-regulated across the two sgRNA-mediated H2AX-KO cell lines (Fig. 3C). The difference in gene expression between the sg1 and sg2 KO samples, as well as the relatively small overlap of differentially expressed genes, likely reflect inherent clonal heterogeneity combined with potential differences in editing efficiency and off-target effects of each sgRNA. Among the down-regulated genes, many are involved in stemness or neurodevelopment, including CXCL12,^35^ KLF4,^36^ JAK3,^37^ and SALL4^38^ (Fig. 3D and E). Pathway analyses revealed that H2AX depletion down-regulated the PI3K-AKT signaling pathway (Fig. S3A and S3B), a critical pathway for GSC maintenance.^39^, ^40^, ^41^ The down-regulation of CXCL12, KLF4, JAK3, and SALL4 was further validated in both U251 and GBM387 cells with H2AX-KO (Fig. 3F and G). These findings collectively indicate that H2AX is essential for the maintenance of GSCs, likely by facilitating the expression of key stemness-associated genes.

### γH2AX is essential for the maintenance of GSCs

**Fig. 4:**
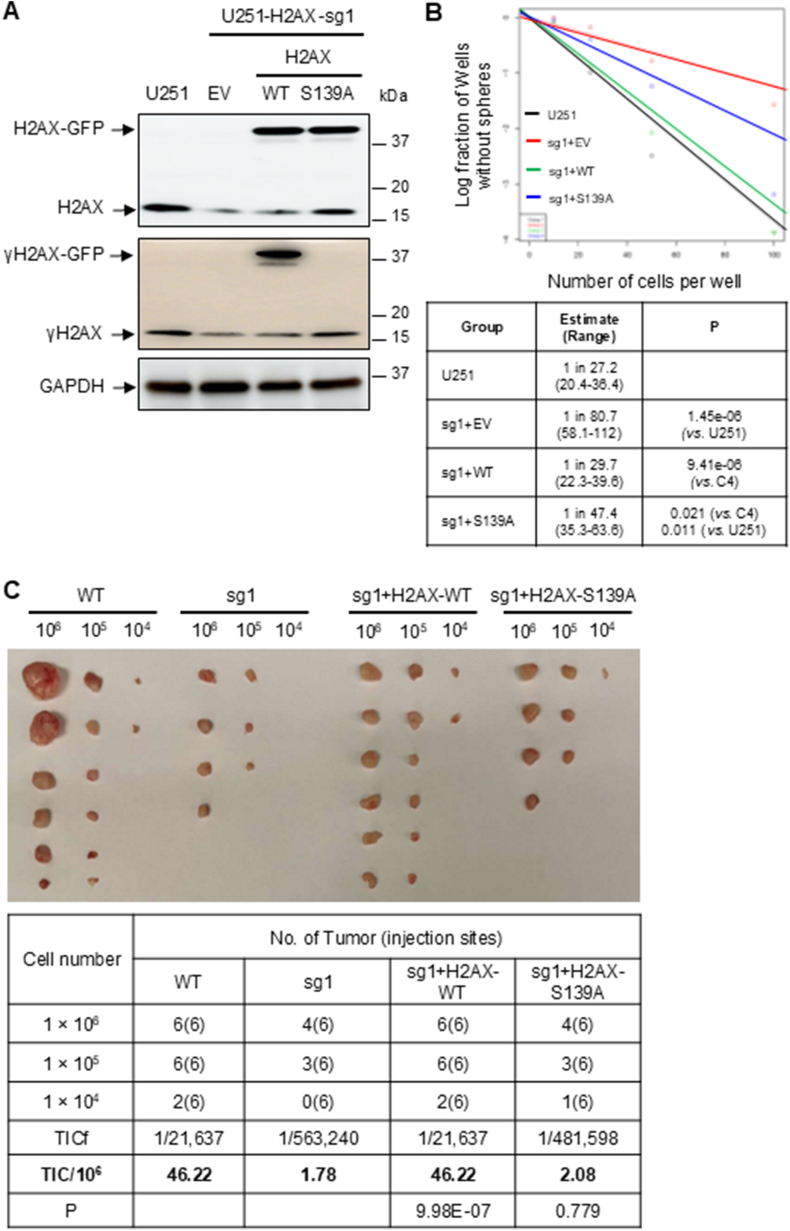
γH2AX is essential for the maintenance of glioma stem cells. **(A)** Restoration of H2AX in H2AX-KO U251 cells. WT or S139A mutant H2AX was transfected into U251-H2AX-sg1 pooled cells. Immunoblotting was conducted to determine the expression of reintroduced H2AX and its corresponding γH2AX. **(B)** Neurosphere formation capacity of H2AX-restored U251-H2AX-KO cells was evaluated using the limiting dilution assay. **(C)** The TICf was estimated using the xenotransplantation limiting dilution assay. Images of xenografts, along with the calculated TICf, are shown.

Given that γH2AX plays a critical role in the maintenance of undifferentiated embryonic stem cells and induced pluripotent stem cells,^18^^,^^21^ and is crucial for reprogramming to pluripotency,^22^ we sought to investigate whether H2AX-mediated maintenance of stemness properties in GBM cells is dependent on γH2AX. To address this, we performed a rescue experiment by stably transfecting GFP-tagged wild-type (WT) or mutant H2AX (S139A) into H2AX-depleted U251 cells (Fig. 4A). The neurosphere formation assay demonstrated that ectopic expression of H2AX-WT restored the self-renewal capacity of U251 cells, whereas expression of H2AX-S139A, in which the phosphorylation site at S139 is abolished, failed to restore the self-renewal ability effectively (Fig. 4B). These cells were injected into nude mice subcutaneously at varying cell number to assess their tumorigenicity. The xenograft assay with limiting dilution revealed a restoration of TICf in H2AX-WT-transfected cells, but not in H2AX-S139A-transfected cells (Fig. 4C). As noted, two tumors in the WT group (10^6^ cell injections) are substantially larger than the others, which may represent outliers resulting from local microenvironmental differences at the implantation site (e.g., vascularization), or technical variation during injection (cell clumping or slight differences in viable cell number). The remaining tumors in this group are comparable in size to those in the sg1 + H2AX-WT group. Importantly, the TICf assay primarily assesses tumor initiation frequency rather than tumor volume, making it unlikely that differences in tumor size affected our TICf estimates. Collectively, these results indicate that phosphorylation of H2AX at S139 is critical to the maintenance of GSCs.

### Increased SET expression contributes to elevated γH2AX levels in GSCs by blocking PP2A activity

**Fig. 5:**
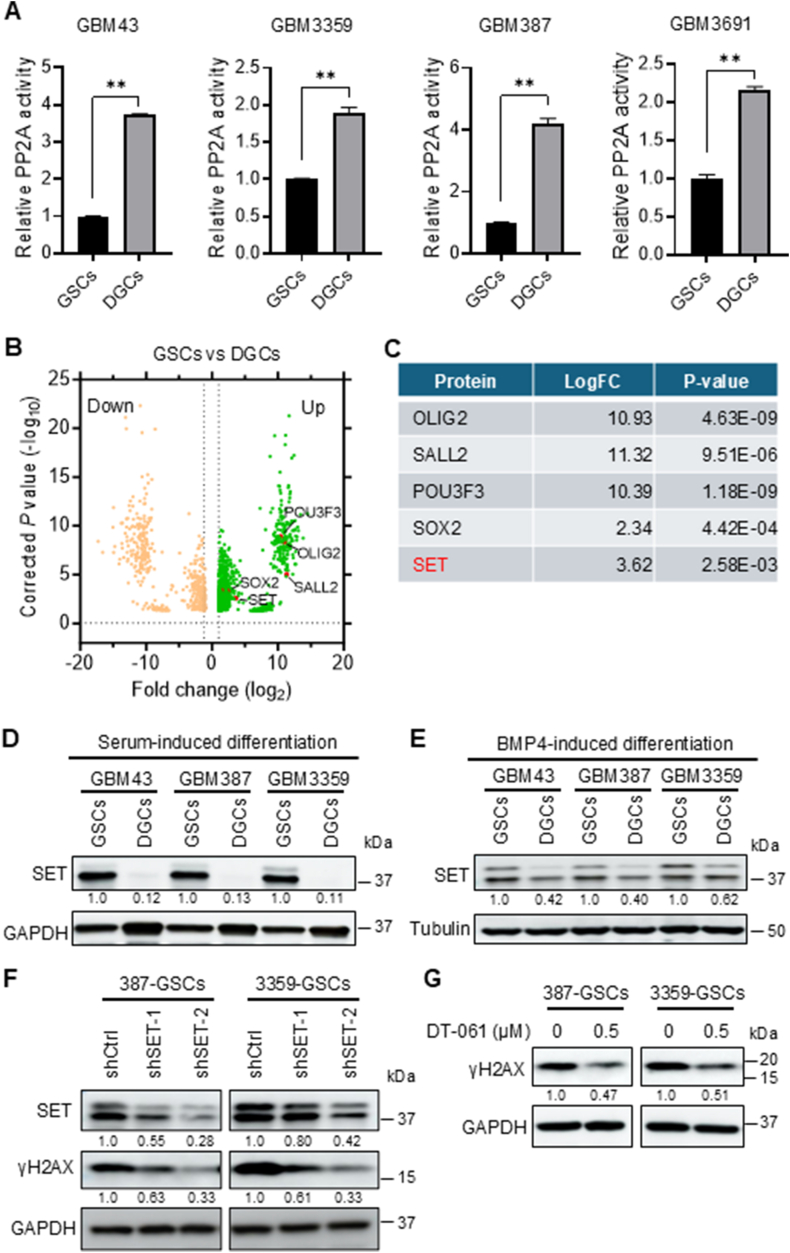
Increased SET expression contributes to elevated γH2AX levels in glioma stem cells (GSCs) by blocking PP2A activity. **(A)** PP2A activity was determined in GBM xenograft-derived GSCs and their corresponding serum-induced differentiated glioma cells (DGCs). **(B, C)** Proteomics analysis demonstrated elevated SET expression in GSCs compared with their corresponding serum-induced DGCs. Spheroid cultured GSCs and serum-induced DGCs derived from GBM387 and GBM3359 cells were subjected to mass spectrometry analysis for identifying differentially expressed proteins (cutoff: |Log fold-change| > 1, *P* < 0.05). The volcano plot illustrates the down-regulated and up-regulated proteins in GSCs compared with DGCs (B). Representative proteins are listed (C). **(D, E)** Immunoblotting was performed to validate the elevated SET expression in GSCs compared with DGCs induced by serum (D) or BMP4 (E). **(F)** The effect of SET knockdown on γH2AX levels in GSCs. GBM387 and GBM3359 cells were transfected with either shCtrl or shSET for 48 h, and immunoblotting was conducted to assess γH2AX levels. **(G)** The effect of PP2A activator DT-061 on γH2AX levels in GSCs. GBM387 and GBM3359 cells were treated with DT-061 (0.5 μM) for 24 h, and immunoblotting was conducted to determine γH2AX levels. Each band was scanned and its intensity quantified; the relative intensity values are shown below the corresponding bands in the figure.

In mammalian cells, γH2AX is typically induced by DNA damage, such as DSBs and replication stress. However, we did not observe an increase in DNA DSBs in GSCs compared with DGCs (Fig. S4A), nor were the DNA damage response and replication stress response significantly activated in GSCs relative to DGCs (Fig. S4B and S4C). H2AX can be phosphorylated by several phosphoinositide 3-kinase-related protein kinases (PIKKs), such as ATM (Ataxia telangiectasia mutated), ATR (ATM and Rad3-related), or DNA-PK (DNA-dependent protein kinase). γH2AX can also be dephosphorylated by protein phosphatase 2A (PP2A).^42^ Thus, the cellular γH2AX level is balanced by the activities of kinases and phosphatases. Given the absence of significant differences between GSCs and DGCs in the factors inducing H2AX phosphorylation, we assessed PP2A phosphatase activity in both cell types. Our results indicate that PP2A activity is significantly lower in GSCs compared with DGCs (Fig. 5A). These results suggest that the elevated γH2AX levels in GSCs are, at least in part, attributable to reduced PP2A activity.

PP2A phosphatase can be negatively regulated by various cellular inhibitory proteins, such as I1PP2A (ANP32A), I2PP2A (SET), CIP2A, ARPP, ENSA, TIP, and PME-1.^43^ To identify potential PP2A inhibitors differentially expressed in GSCs compared with DGCs, we performed proteomic analyses on GSCs and DGCs derived from GBM387 and GBM3359 cell lines. We identified 898 up-regulated and 496 down-regulated proteins (|Log fold-change| > 1, *P* < 0.05) in both types of GSCs compared with their corresponding DGCs (Fig. 5B; Table S2). Among the up-regulated proteins, we identified OLIG2, SALL2, POU3F3, and SOX2, all well-established stemness markers of GSCs, confirming the accuracy of our quantitative proteomics analyses (Fig. 5B and C). Notably, the SET protein, a known PP2A inhibitor, was significantly elevated in GSCs compared with DGCs (Fig. 5B and C). Western blotting analysis further validated the increased SET protein expression in multiple GSC populations, relative to their respective DGC counterparts, induced by either serum or BMP4 differentiation (Fig. 5D and E). To investigate whether elevated SET protein contributes to increased γH2AX in GSCs, we knocked down SET in GBM387 and GBM3359 GSCs and found that down-regulation of SET indeed reduces the level of γH2AX (Fig. 5F). We further treated GBM387 and GBM3359 GSCs with a PP2A activator DT-061, and we found that PP2A activation significantly reduced γH2AX levels (Fig. 5G). Collectively, these findings suggest that elevated SET expression in GSCs contributes to increased γH2AX levels by inhibiting PP2A activity.

## Discussion

The maintenance of stemness properties in tumor cells, including GBM, is governed by a core set of transcription factors and special chromatin structure. Histone variants contribute to the chromatin landscape with specific functions, and their dysregulation has been linked to the initiation and progression of cancer.^44^ Additionally, histone variants such as H2AX and H3.3 are believed to play essential roles in preserving stemness in stem cells and promoting somatic cell reprogramming to a pluripotent state by regulating gene expression.^45^ However, the role of histone variants in the maintenance of GSCs remains unclear. In this study, we show that the histone variant H2AX, along with its phosphorylated form γH2AX, is up-regulated in GSCs compared with their corresponding DGCs. Both H2AX and γH2AX play critical roles in the maintenance of the self-renewal and tumor-initiating capability in GBM cells.

H2AX is an important histone variant that plays multiple roles in cellular processes, including DDR and epigenetic regulation.^20^^,^^46^ The epigenetic regulatory function of H2AX and its phosphorylated form (γH2AX) has been implicated in the maintenance of undifferentiated embryonic stem cells and induced pluripotent stem cells,^18^^,^^21^ as well as during the reprogramming to pluripotency.^22^ In pluripotent cells, high basal γH2AX levels are thought to be associated with global chromatin decompaction rather than pre-existing DNA damage and have been suggested to play an important role in the maintenance of the pluripotent state.^20^, ^21^, ^22^ γH2AX is also thought to function as a transcriptional regulator, with enrichment observed at pluripotency gene loci but not ectoderm- or mesoderm-related gene loci in human pluripotent stem cells. However, the exact mechanisms by which γH2AX modulates gene transcription remain unclear and warrant further investigation. Based on our findings in this study, we hypothesize that H2AX/γH2AX modulates local chromatin structure at pluripotency and differentiation gene loci in GBM cells, thereby sustaining stemness traits and promoting dedifferentiation. In the future study, we will further identify genes that are directly regulated by H2AX and γH2AX in GSCs and assess the effect of H2AX and γH2AX on chromatin accessibility and states in pluripotent and differentiation genes in GSCs.

It is well known that pluripotency can be induced in fully differentiated somatic cells with four transcription factors: OCT4, KLF4, SOX2, and MYC (OKSM, Yamanaka factors).^36^ It is reported that the OKSM factors cooperate with γH2AX to accelerate reprogramming. Specifically, three out of four OKSM factors, OCT4, SOX2, and MYC, but not KLF4, cooperate to induce γH2AX during reprogramming to pluripotency.^22^ Our study further demonstrated that H2AX depletion significantly reduces the KLF4 expression but not SOX2, OCT4, and MYC expression (Table S1), indicating that among the four OKSM factors, KLF4 might be the downstream gene regulated by H2AX/γH2AX deposition. Given that KLF4 is best known for its role in maintaining the pluripotency of embryonic stem cells and induction of reprogramming to pluripotency, it is possible that H2AX/γH2AX deposition facilitates the maintenance of stemness features in GSCs and promotes dedifferentiation of DGCs by up-regulating KLF4 expression. However, we cannot rule out the possibility that H2AX/γH2AX directly regulates other stemness-related genes, as our RNA-sequencing analysis revealed the down-regulation of multiple stemness-related genes in H2AX KO GBM cells.

We have demonstrated that the γH2AX level is drastically higher in GSCs compared with DGCs. We also found that the γH2AX level increases after dedifferentiation of DGCs. Normally, H2AX can be phosphorylated by ATM, ATR, and DNA-PK upon DNA damage formation.^47^, ^48^, ^49^ Endogenous DNA damage and replication stress, driven by cellular metabolism and proliferation, can lead to constitutive γH2AX accumulation. However, this phosphorylation is counteracted by dephosphorylation through phosphatases such as PP2A and PP4C,^42^^,^^50^, ^51^, ^52^ maintaining low γH2AX levels in the absence of exogenous stress. Therefore, the accumulation of cellular γH2AX under normal cultured conditions may result from reduced dephosphorylation. Our findings reveal that GSCs exhibit diminished PP2A activity compared with DGCs. Notably, reactivation of PP2A in GSCs leads to a reduction in γH2AX levels, indicating that impaired PP2A activity is a key factor contributing to the elevated γH2AX observed in GSCs.

PP2A is a highly conserved, ubiquitously expressed Serine/Threonine phosphatase that constitutes a significant portion of the total phosphatase activity in eukaryotic cells.^53^ It is widely recognized as a tumor suppressor due to its critical role in negatively regulating key signaling pathways that govern cell growth, survival, and differentiation. PP2A also plays a critical role in regulating stemness through diverse, context-dependent mechanisms that vary across stem cell and cancer types.^54^ In certain cancers, PP2A inhibition promotes CSC expansion by modulating key signaling pathways, such as AKT/mTOR and Wnt/β-catenin.^54^^,^^55^, Conversely, PP2A can promote CSC expansion by suppressing cellular differentiation or enhancing symmetric cell division. In glioma, PP2A inhibition induces differentiation by disrupting the nuclear receptor corepressor (N-CoR) complex, which is essential for maintaining neural stem cells and GBM cells in an undifferentiated state.^56^ In contrast, increased PP2A activity through its interaction with MUC1 has been shown to drive the expansion of cancer stem-like cells in small cell lung cancer by shifting the balance toward symmetric cell division.^57^ In this study, our data suggest that inactivation of PP2A may enhance the stemness properties of GSCs by promoting γH2AX accumulation, unveiling a previously unrecognized mechanism by which PP2A suppresses cancer stem cell characteristics. However, further investigation is necessary to validate the role of the PP2A–γH2AX axis in maintaining GSC stemness, which will be addressed in future studies.

PP2A has been shown to be genetically altered or functionally inactivated in many solid cancers and leukemia, as well as CSCs.^55^^,^^58^ PP2A is a heterotrimeric enzyme composed of a catalytic C subunit, a structural A subunit, and a regulatory B subunit. The substrate specificity and functional activity of PP2A are dictated by the incorporation of specific regulatory B subunit families, including B55, B56, PR72, and PR93/PR110.^53^ In addition, PP2A activity can be regulated by endogenous PP2A inhibitors, such as SET, CIP2A, PME-1, or ENSA/ARPP19.^43^ Our proteomics analyses did not identify significant alterations in the expression levels of PP2A subunits (Table S2). However, we observed a marked increase in SET protein levels in GSCs compared with DGCs. SET oncoprotein, also known as I2PP2A, is a potent physiologic inhibitor of PP2A.^59^ SET protein directly binds to PP2Ac through its N-terminus and C-terminus regions, and inhibits PP2A phosphatase activity.^60^ SET is ubiquitously expressed across various human tissues, including the kidney, liver, brain, spleen, lung, heart, and gonadal system. Its overexpression has been implicated in the development and progression of numerous cancers,^61^ primarily through the formation of an inhibitory complex with PP2A, thereby amplifying pro-growth and pro-survival signaling pathways associated with tumor progression. SET has been reported to play a role in maintaining pluripotency in embryonic stem cells.^62^^,^^63^ SET-mediated PP2A inactivation is also essential for the self-renewal of leukemic stem cells. Pharmacological inhibition of SET eradicates tyrosine kinase inhibitor (TKI)-resistant chronic myeloid leukemia stem cells and KMT2A-rearranged acute myeloid leukemia stem cells by reactivating PP2A, which in turn suppresses β-catenin signaling and down-regulates MYC expression.^64^^,^^65^ Our findings reveal that elevated SET expression in GSCs drives γH2AX accumulation, a critical factor for maintaining GSC properties. Notably, down-regulation of SET reduces γH2AX levels, highlighting a novel mechanism through which the SET-PP2A axis promotes cancer stemness and contributes to glioblastoma progression.

## Conclusions

**Fig. 6:**
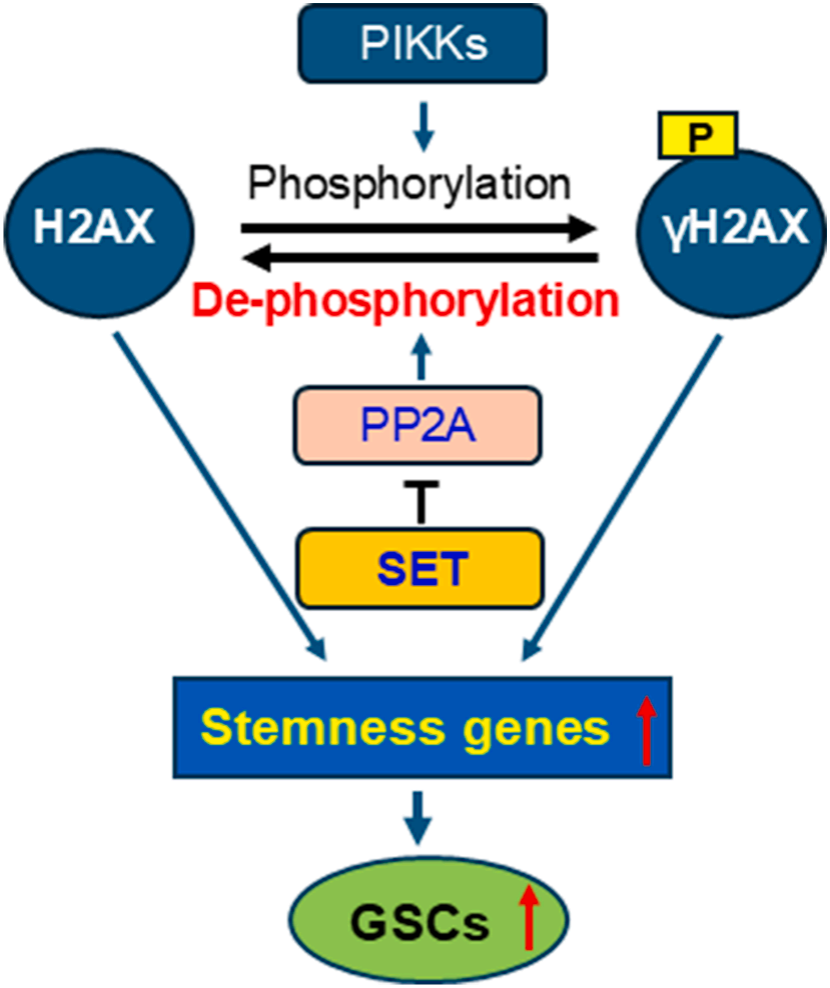
Schematic illustration of the regulation of H2AX phosphorylation and its role in maintaining glioma stem cells (GSCs). Up-regulated SET expression suppresses PP2A activity, resulting in the accumulation of γH2AX due to phosphatidylinositol 3-kinase-related kinases (PIKKs) activation in response to endogenous DNA damage. Increased γH2AX levels drive the transcription of stemness-associated genes, thereby supporting the self-renewal and maintenance of GSCs.

This study offers significant biological insights into the regulation of stemness traits in GSCs. We demonstrated that the histone variant H2AX and its phosphorylated form, γH2AX, are highly expressed in GSCs and play a pivotal role in maintaining their stem-like properties. The elevated γH2AX levels are primarily driven by SET-mediated inhibition of PP2A phosphatase activity (Fig. 6). Thus, reducing γH2AX, potentially through PP2A reactivation, represents a promising therapeutic strategy to disrupt GSC stemness and mitigate tumor recurrence in glioblastoma patients. Future studies will focus on validating the role of H2AX in GBM stemness using patient-derived data and primary tumor samples, as well as elucidating the detailed mechanisms by which H2AX phosphorylation regulates GBM stemness.

## CRediT authorship contribution statement

**Chunhua Han:** Writing – review & editing, Writing – original draft, Methodology, Investigation, Conceptualization. **Na Li:** Methodology, Investigation. **Ananya Banerjee:** Methodology, Investigation. **Linzhou Wang:** Investigation. **Yajing Yang:** Investigation. **Yaogang Zhong:** Resources, Investigation. **Sophia Wu:** Investigation. **Heather Manring:** Investigation. **Nan Zhang:** Investigation. **Christian A. Showalter:** Investigation. **Karnika Singh:** Investigation. **Zhen-Yue Tong:** Investigation. **Xiaomei Meng:** Investigation. **Zaid Sirhan:** Investigation. **Jaharul Haque:** Investigation. **Qingfei Zheng:** Investigation. **Monica Venere:** Resources, Investigation. **Deliang Guo:** Resources, Investigation. **Qi-En Wang:** Writing – review & editing, Writing – original draft, Supervision, Funding acquisition, Formal analysis, Conceptualization. **Arnab Chakravarti:** Writing – review & editing, Writing – original draft, Supervision, Project administration, Funding acquisition, Conceptualization.

## Funding

This work was supported by the 10.13039/100000054National Cancer Institute (USA) (No. R01CA169368 to A.C., R01CA188228 to A.C., R01CA227874 to D.G. and A.C., UG1CA233331 to A.C., and P30CA016058).

## Conflict of interests

The authors declare no conflict of interests.
