# Supplementary material for: H2AX phosphorylation driven by the SET-PP2A axis maintains glioma stem cell properties
Source: Genes Dis. 2025 Dec 2;13(6):101961. doi: 10.1016/j.gendis.2025.101961 (PMC13474088; doi:10.1016/j.gendis.2025.101961)
Supplement: Supplementary file 1 [file mmc1.docx]

**Supplementary Figures**

**Elevated H2AX promotes stemness of glioma stem cells**

Chunhua Han^1^, Na Li^1^, Ananya Banerjee^1^, Linzhou Wang^1^, Yajing Yang^1^, Yaogang Zhong^1^, Sophia Wu^1,^ ^2^, Heather Manring^1^, Nan Zhang^1^, Christian A. Showalter^1^, Karnika Singh^1^, Zhen-Yue Tong^1^, Xiaomei Meng^1^, Zaid Sirhan^1^, Jaharul Haque^1^, Qingfei Zheng^1^, Monica Venere^1^, Deliang Guo^1^, Qi-En Wang^1,^*, Arnab Chakravarti^1,^ *

^1^Department of Radiation Oncology, Comprehensive Cancer Center, The Ohio State University, Columbus, OH 43210

^2^Columbus Academy, Gahana, OH 43230

*: Corresponding author

**Supplementary Fig. 1 Glioma stem cells possess increased base levels of H2AX and γH2AX. A, B** Immunoblotting was conducted to evaluate the expression levels of H2AX and γH2AX in GBM xenograft-derived GSCs from GBM3691 (A) and GBM10 (B) models, as well as their corresponding DGCs induced by FBS. **C-F** GSCs and DGCs generated from xenograft-derived GBM387 (C, D) and GBM3359 cells (E, F) were subjected to immunofluorescence to assess the expression of γH2AX. The number of γH2AX foci per nucleus was counted in over 40 cells for each sample and plotted (D, F). Specifically, 48 GBM387 cells and 42 GBM3359 cells were analyzed. *: p < 0.05; **: p < 0.01.

**Supplementary Fig. 2 H2AX is critical to the maintenance of GSCs. A, B** Representative images of neurospheres derived from GBM387 (A) and U251 (B) cells with H2AX-KO. **C, D** Images of the xenografts derived from H2AX-KO GBM387 (C) and U251 (D) cells. **E, F** The TICf was estimated in LN229 cells and H2AX-KO LN229 cells using a xenotransplantation limiting dilution assay. Immunoblotting confirms H2AX KO (E); Images of xenografts derived from LN229 cells, along with the calculated TICf (F).

**Supplementary Fig. 3 H2AX depletion impairs the PI3K-AKT signaling pathway. A** Functional enrichment analysis was conducted in common downregulated genes shared between the sg1 and sg2 groups using DAVID. **B.** Gene Set Enrichment Analysis (GSEA) indicates a negative correlation between H2AX-KO and the PI3K-AKT signaling pathway in both sg1 and sg2 groups.

**Supplementary Fig. 4 Elevated γH2AX levels are not attributed to increased endogenous DNA damage in GSCs. A** The Comet Assay was conducted to evaluate DNA DSBs in GSCs and DGCs. DGCs were irradiated with X-rays (1 Gy, 1 h) to serve as positive controls. **B** Immunoblotting was conducted to assess the expression levels of DNA damage response (DDR) proteins in GBM xenograft-derived GSCs and their corresponding serum-induced DGCs, as well as in GBM cell lines and their corresponding spheroid culture-enriched GSCs. **C** Immunoblotting was conducted to assess the levels of phosphorylated RPA32 (pRPA32) in GBM xenograft-derived GSCs and their corresponding serum-induced or BMP4-induced DGCs.
